# Ultramorphological Comparison of Proboscis and Associated Sensilla of *Scotogramma trifolii* and *Protoschinia scutosa* (Lepidoptera: Noctuidae)

**DOI:** 10.3390/insects12110992

**Published:** 2021-11-04

**Authors:** Chuan-Min Zhang, Yue Niu, Gui-Lin Hu, Ji-Qi Lu

**Affiliations:** Institute of Biodiversity and Ecology, School of Life Sciences, Zhengzhou University, Zhengzhou 450001, China; zcmin@gs.zzu.edu.cn (C.-M.Z.); Niuy2020@163.com (Y.N.)

**Keywords:** feeding mechanisms, flower visitor, mouthparts, scanning electron microscopy, sensory organ

## Abstract

**Simple Summary:**

The clover cutworm, *Scotogramma trifolii* Rottemberg, and the spotted clover moth, *Protoschinia scutosa* (Denis & Schiffermuller), are worldwide polyphagous pests, and the larvae feed mainly on the leaf backs of many agricultural crops. However, the food sources and feeding habits of the adults are still poorly known. We investigated the ultramorphology of the proboscis and associated sensilla of *S. trifolii* and *P. scutosa* using scanning electron microscopy. The results show that the proboscises of *S. trifolii* and *P. scutosa* are structurally similar, both including three sensillum types and three zones (Zone 1–3). The sensillum chaeticum is non-porous hair-like, the sensillum basiconicum is a short smooth cone with a sensory pore on the blunt tip, and each sensillum styloconicum is composed of a uniporous sensory cone inserted into a ribbed stylus. In addition, the movement and fluid uptake mechanisms of the proboscis and the possible function of sensilla are briefly discussed.

**Abstract:**

The proboscis is an important feeding organ for the glossatan moths, mainly adapted to the flower and non-flower visiting habits. The clover cutworm, *Scotogramma trifolii* Rottemberg, and the spotted clover moth, *Protoschinia scutosa* (Denis & Schiffermuller), are serious polyphagous pests, attacking numerous vegetables and crops, resulting in huge economic losses. However, the feeding behavior and mechanisms of the adult stage remain unsatisfactorily explored. In this study, the proboscis morphology of *S. trifolii* and *P. scutosa* are described in detail using scanning electron microscopy, with the aim of investigating the morphological differences and feeding behavior of these two species. The proboscises of *S. trifolii* and *P. scutosa* are similar in morphology and structure and are divided into three zones (Zone 1–3) based on the morphological changes of the dorsal legulae. Three sensillum types are located on the proboscises of both species, sensilla chaetica, sensilla basiconica, and sensilla styloconica. Significant differences were observed in the length of the proboscis and each zone between these two species, as well as in sensilla size and number. Based on the morphology of the proboscis and associated sensilla, *S. trifolii* and *P. scutosa* are potential flower visitors, which was also reinforced by the pollen observed at the proboscis tip. These results will strengthen our understanding of the structure of the proboscis related to the feeding behavior of Noctuidae.

## 1. Introduction

Insect mouthparts are modified appendages of head segments, bearing various types of sensory organs and adapted to exploit different food resources [1]. Due to host selection and optimized feeding techniques, the structure of the mouthparts has undergone significant differentiation in the long evolutionary process [2,3]. Characteristic adaptations of mouthparts have resulted in feeding specialization and enhanced functional performance [3]. For example, the chewing mouthparts in Orthoptera [4] and Coleoptera [5,6,7] have been adapted for grinding, chewing, pinching, or crushing bits of solid food, whereas the piercing–sucking mouthparts in Hemiptera [8,9,10] have evolved to feed on the tissue of the host plants [11]. In Lepidoptera, a siphoning type of proboscis has evolved only once, and is an autapomorphy of the Glossata [1]. The proboscis is well-suited to feed on nectar, pollen, fruit, blood and tears, mainly adapted to flower and non-flower visiting habits [1,12].

In adult Glossata, the proboscis has a relatively simple morphology, but it is unique in its coiled resting position. The proboscis consists of two extended concave maxillary galeae and a hollow food tube, which are joined by dorsal and ventral cuticular projections, i.e., legulae [13,14]. The proboscis with specialized structural organization probably has co-evolved in context with the flowering plant in the Cretaceous [1], thus facilitating the diversification of feeding habits in Glossata [15]. An array of mechano- and chemosensilla on the proboscis of Glossatan species, such as sensilla chaetica, basiconica, and styloconica, play important roles in host localization and feeding [16,17,18]. Previous studies on morphology of proboscis sensilla in Glossata have concentrated mainly on Noctuidae [12,16,19,20], and Nymphalidae [21,22,23,24].

Noctuidae (*sensu stricto*) is the second largest family in Lepidoptera, including numerous agricultural pests of great economic significance [25,26]. The clover cutworm, *Scotogramma trifolii* Rottemberg, and the spotted clover moth, *Protoschinia scutosa* (Denis & Schiffermuller), are worldwide polyphagous pests in the subfamily Noctuinae and Heliothinae, respectively [27,28]. Adults of *S. trifolii* and *P. scutosa* have characteristics of intermittent local outbreaks and migration [29,30]. The larvae of *S. trifolii* and *P. scutosa* feed mainly on many agricultural crops, such as *Gossypium herbaceum* Linnaeus [31,32,33] and *Chenopodium quinoa* Willd [34], while the food sources and feeding habits of the adults still remain poorly known. The morphology of proboscis and associated sensilla could provide insights into adult feeding behavior and the mechanisms of *S. trifolii* and *P. scutosa*, but it has never been satisfactorily explored.

In this study, we investigated the ultramorphology of the proboscis and associated sensilla of *S. trifolii* and *P. scutosa* using scanning electron microscopy, with the aim of interpreting the function of the feeding apparatus and exploring the feeding behavior of these two noctuid species. This study also made morphological comparisons between *S. trifolii* and *P. scutosa* and between sexes within species, including the distribution, number and dimension of the proboscis sensilla. These findings provide a morphological basis to understand the feeding mechanisms of noctuid species.

## 2. Materials and Methods

### 2.1. Specimen Sampling

Adults of *S. trifolii* and *P. scutosa* were obtained from the Modern Agricultural Science and Technology Demonstration Base of Henan Academy of Agricultural Sciences, Yuanyang County, Henan Province, China (35°00′ N, 113°41′ E). The proboscis was cut from the head with dissecting scissors under a stereomicroscope (SZN71, Sunny Optical Technology Co., Ltd., Ningbo, China). The samples were fixed in Carnoy’s fixative solution (95% ethanol: glacial acetic acid = 3:1) for 24 h, and then immersed in 75% ethanol solution.

### 2.2. Scanning Electron Microscopy (SEM)

The proboscis was washed using an ultrasonic cleaner (KQ118, Kunshan, China) for a few seconds. All samples were dehydrated through a graded ethanol series of 80%, 85%, 90%, and 95% for 10–15 min each, and 100% for 30 min, twice. The proboscis was naturally dried on filter paper. The dried samples were mounted at various angles using double-sided graphite adhesive tape, and sputter coated with gold. All samples were examined under a Hitachi S-3400N scanning electron microscope (Hitachi, Tokyo, Japan) at 5.00 kV and full vacuum.

### 2.3. Statistical Analysis

The proboscis dimensions and sensilla size were measured using Imaris 7.4.2 software with seven males and seven females of each species. An independent sample *t*-test was used to evaluate differences of proboscis and associate sensilla between species and sexes (*p* < 0.05) in SPSS Statistics v 22.0.0.0. The software Photoshop CS (Adobe, San Jose, CA, USA) was used to adjust contrast and levels of SEM images.

### 2.4. Terminology

Morphological descriptions of the proboscis follow the terminology of Lehnert et al. [35] and Faucheux [16]. The proboscis was structurally delineated into three zones (Zone 1–3). These zones were determined and defined by Lehnert et al. [22]. Zone 1 is hydrophobic and longest among the three zones; Zone 2 is the main hydrophilic zone, allowing liquid to enter the food canal through interlegular spaces; Zone 3 occupies only a small portion of the tip of the proboscis, without dorsal legulae.

## 3. Results

### 3.1. General Morphology of Proboscis

**Figure 1:**
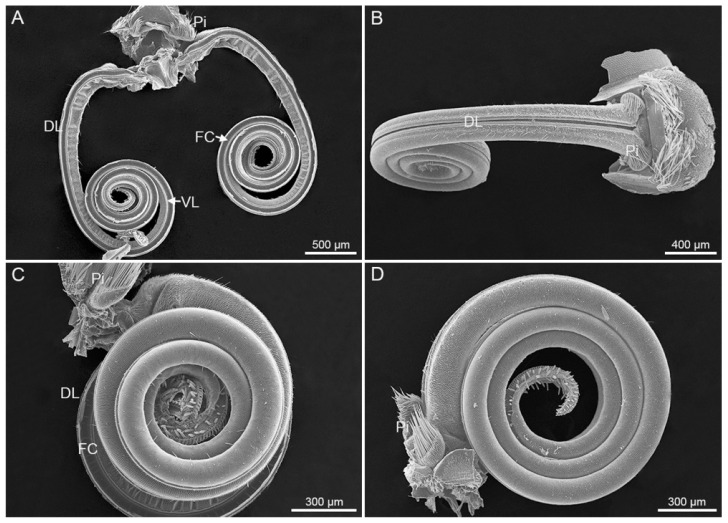
The proboscis morphology. (**A**) General interior view of *Scotogramma trifolii*; (**B**) dorsal view of *Protoschinia scutosa*; (**C**) lateral view of *S. trifolii*; (**D**) lateral view of *P. scutosa*. DL, dorsal legulae; FC, food canal; Pi, pilifer; VL, ventral legulae.

**Figure 2:**
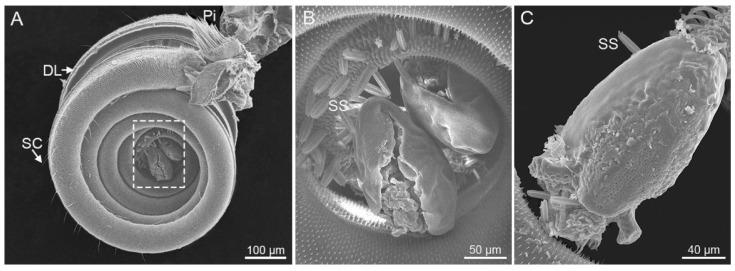
The proboscis of *Protoschinia scutosa*. (**A**) Lateral view; (**B**) enlarged view of the dotted rectangle in (**A**); (**C**) pollen grain on the proboscis tip. DL, dorsal legulae; Pi, pilifer; SC, sensilla chaetica; SS, sensilla styloconica.

The proboscises of *S. trifolii* and *P. scutosa* are nearly same in shape and structure, both consisting of two elongated and coiled maxillary galeae (Figure 1A,B). The maxillary galeae are tightly linked by a series of dorsal and ventral legulae, thus generating a sucking tube. The proboscis is coiled into 4–5 turns in the resting position, and the coils are closely connected (Figure 1C,D). The lateral lobe and pilifer bear long bristles at the base of the proboscis. Floral pollens were wrapped at the tip region of the coiled proboscis in *P. scutosa* (Figure 2). The length of the proboscis is similar for *S. trifolii* and *P. scutosa* (*p* > 0.05) (Table 1).

**Figure 3:**
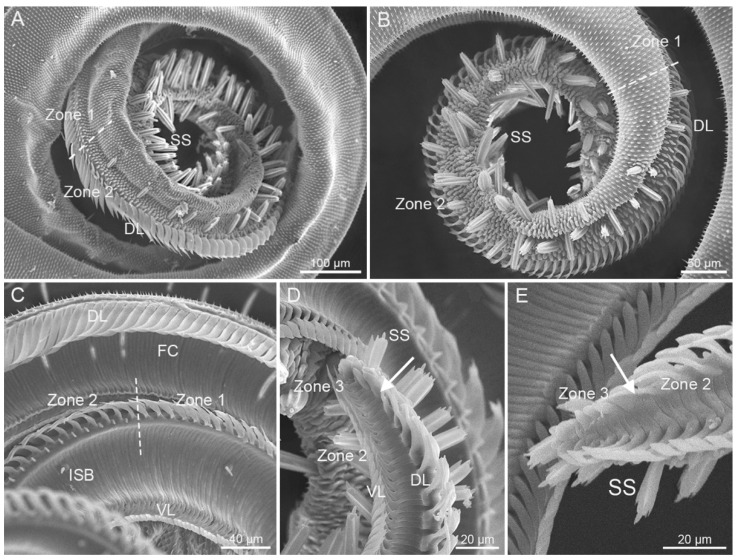
The division of proboscis. (**A**) External view of the boundary between Zones 1 and 2 of *S. trifolii*; (**B**) external view of the boundary between Zones 1 and 2 of *P. scutosa*; (**C**) inner view of the boundary between Zones 1 and 2 of *S. trifolii* (indicated by white dashed line); (**D**) inner view of the boundary between Zones 2 and 3 of *S. trifolii* (indicated by white arrow); (**E**) inner view of the boundary between Zones 2 and 3 of *P. scutosa* (indicated by white arrow). DL, dorsal legulae; ISB, internal sensilla basiconica; SS, sensilla styloconica; VL, ventral legulae.

According to the shape, the size, and the absence of the dorsal legulae, the proboscis can be structurally delineated into three zones (Zone 1–3), which are present on both *S. trifolii* and *P. scutosa* (Figure 3). Zone 1 is characterized by tightly linked lancet-shaped dorsal legulae, whereas Zone 2 is unique in having wider dorsal legulae overlapping in a shingle-like fashion (Figure 3A–C). Zone 3 is the most distal and the smallest region at the apex, without dorsal legulae (Figure 3D,E). The rough external surface is equipped with transverse cuticular processes on Zone 1 and numerous microbumps on Zone 2 and 3, respectively. The concave inner surface of proboscis bears closely connected smooth transverse ridges (Figure 3C). *S. trifolii* exhibit significant differences from *P. scutosa* in the length of each zone, as well as in the size of sensilla (Table 1 and Table 2). No significant differences are found between sexes within each species (Table 3 and Table 4). *P. scutosa* has a markedly longer Zone 1 and 3 than *S. trifolii* (Table 1).

#### 3.1.1. Zone 1

**Figure 4:**
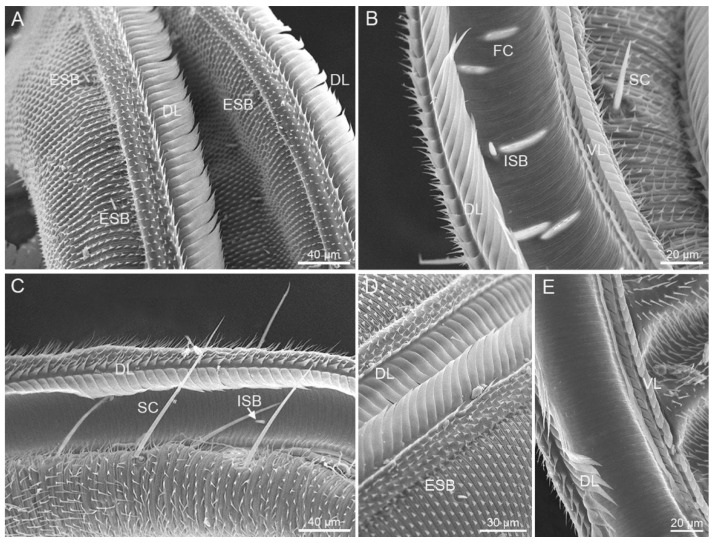
Zone 1. (**A**) Dorsal view of *S. trifolii*; (**B**) internal view of *S. trifolii*; (**C**,**E**) internal view of *P. scutosa*; (**D**) dorsal view of *P. scutosa*. DL, dorsal legulae; ESB, external sensilla basiconica; FC, food canal; ISB, internal sensilla basiconica; SC, sensilla chaetica; VL, ventral legulae.

Numerous triangular microtrichia are arranged in regular rows on the galeal surface of Zone 1 (Figure 4A), and gradually become shorter towards Zone 2. The surface of the food canal is composed of horizontal grooves which are tightly arranged in parallel (Figure 4B,C,E). The diameter of the food canal on Zone 1 remains almost constant, and shows non-significant differences between *S. trifolii* and *P. scutosa* (Table 1). The dorsal legulae of both species are smooth lancet-shaped plates, arranged horizontally, and overlapped with the opposite (Figure 4D). The ventral legulae include two rows of flattened plates, with the internal row narrower and shorter than the external (Figure 4E). The flattened plates are tightly connected. *S. trifolii* and *P. scutosa* have a mean length of 6.43 ± 0.17 mm and 7.16 ± 0.16 mm on Zone 1, occupying more than 80% and 90% of the total proboscis, respectively (Table 1). The length of Zone 1 is significantly different between these two species, but similar between sexes within species (Table 3 and Table 4). Zone 1 of both species bears sensilla chaetica and sensilla basiconica on the external surface, and sensilla basiconica on the food canal (Figure 4).

#### 3.1.2. Zone 2

**Figure 5:**
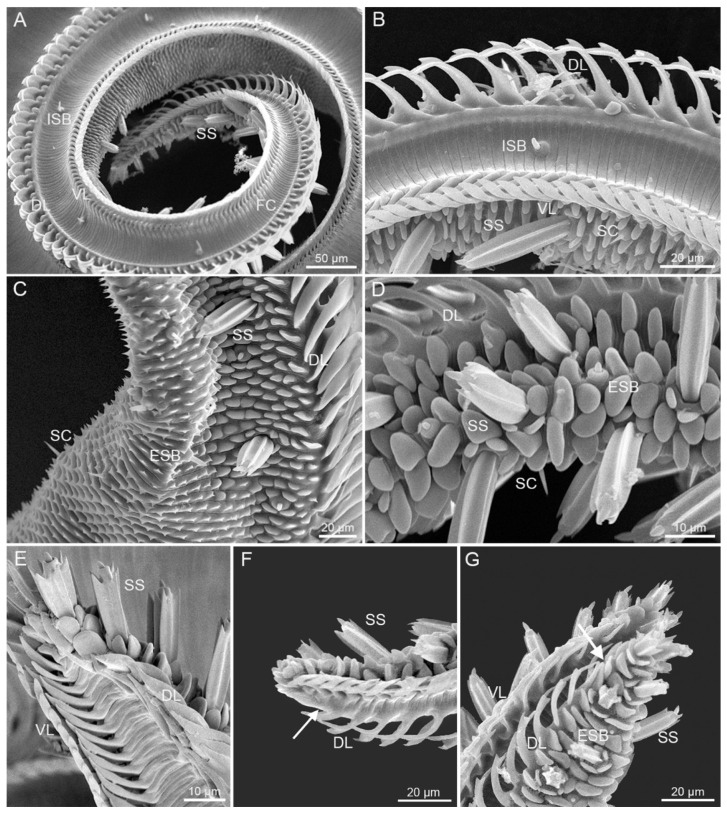
Zones 2 and 3. (**A**) Internal view of Zone 2 of *S. trifolii*; (**B**) internal view of Zone 2 of *P. scutosa*; (**C**) external view of Zone 2 of *S. trifolii*; (**D**) external view of Zone 2 of *P. scutosa*; (**E**) internal view of Zones 2 and 3 of *S. trifolii*; (**F**) internal view of Zone 3 of *S. trifolii*, with the white arrow indicating the boundary between Zones 2 and 3; (**G**) lateral view of Zone 3 of *P. scutosa*, with the white arrow indicating the boundary between Zones 2 and 3. DL, dorsal legulae; ESB, external sensilla basiconica; FC, food canal; ISB, internal sensilla basiconica; SC, sensilla chaetica; SS, sensilla styloconica; VL, ventral legulae.

The galeal surface on Zone 2 possesses microbumps of various shapes and sizes (Figure 5C,D). Around six microbumps surround the sensilla styloconica, and form a lotus-shaped base. The food canal on Zone 2 is morphologically similar to those on Zone 1, progressively tapering toward the tip (Figure 5A,B). The dorsal legulae on Zone 2 are parallel to each other and nearly perpendicular to the cross section of galea, leaving some slits between the dorsal legulae. Each dorsal legula is curved in a sickle shape toward the tip, widened at the base and bifurcated at the apex. The ventral legulae on Zone 2 consist of two rows of flattened plates, and the internal row is much narrower than the external, with enlarged interlegular space (Figure 5E). *S. trifolii* has a significantly longer Zone 2 than *P. scutosa* (Table 1). No significant differences are observed in the length of Zone 2 between sexes within species (Table 3 and Table 4). Zone 2 possesses three types of sensilla: sensilla chaetica, sensilla basiconica, and sensilla styloconica (Figure 5A–D).

#### 3.1.3. Zone 3

Zone 3 is the distalmost zone without dorsal legulae on the proboscis of both species (Figure 5E,F). The external surface of Zone 3 is covered with blunt microbumps. The internal surface is morphologically similar to that of Zone 1 and 2, but with a much narrower food canal. The ventral legulae on Zone 3 consist of two rows of flattened plates, nearly same to those on Zone 2 (Figure 5E). Zone 3 of *S. trifolii* is significantly shorter than that of *P. scutosa* (Table 1). No significant differences are found in the length of Zone 3 between sexes within species (Table 3 and Table 4). Two types of sensilla are visible on Zone 3 of both species: sensilla basiconica and sensilla styloconica.

### 3.2. Proboscis Sensilla

#### 3.2.1. Sensilla Chaetica

**Figure 6:**
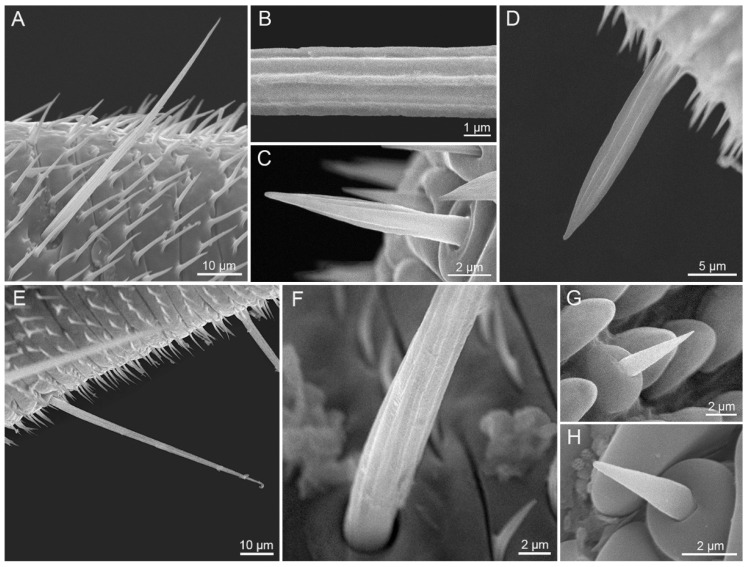
Sensilla chaetica. (**A**,**B**,**D**) Sensillum chaeticum on Zone 1 of *S. trifolii*; (**C**,**E**,**F**) sensillum chaeticum on Zone 1 of *P. scutosa*; (**G**) sensillum chaeticum on Zone 2 of *S. trifolii*; (**H**) sensillum chaeticum on Zone 2 of *P. scutosa*.

Sensilla chaetica are distributed on the galeal surface of Zones 1 and 2. Sensillum chaeticum is non-porous hair-like, and gradually tapers toward the tip (Figure 6). The base of sensillum chaeticum is embedded in a concave pit, standing nearly perpendicularly to the cuticle surface. Sensilla chaetica on Zone 1 are elongated and equipped with longitudinal striations (Figure 6A–G), but become short and smooth on Zone 2 (Figure 6H). The most abundant sensilla, sensilla chaetica, are on the proboscis of both species, which are denser at the basal and much sparser on the distal part. Sensilla chaetica of *P. scutosa* are significantly longer and wider than those of *S. trifolii* (Table 2). Significant differences are observed in the width of sensilla chaetica between sexes within each species (Table 3 and 4).

#### 3.2.2. Sensilla Basiconica

**Figure 7:**
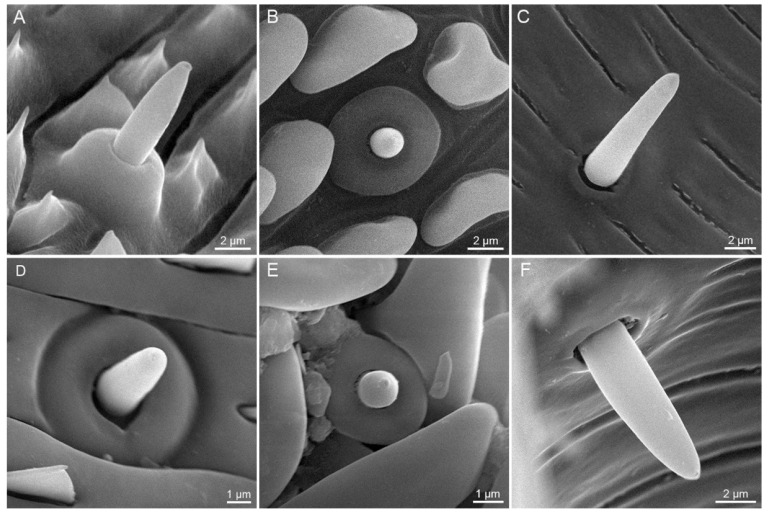
Sensilla basiconica. (**A**) External sensillum basiconicum on Zone 1 of *S. trifolii*; (**B**) external sensillum basiconicum on Zone 2 of *S. trifolii*; (**C**) internal sensillum basiconicum on food canal of *S. trifolii*; (**D**) external sensillum basiconicum on Zone 1 of *P. scutosa*; (**E**) external sensillum basiconicum on Zone 2 of *P. scutosa* showing a sensory pore at the blunt tip; (**F**) Internal sensillum basiconicum on food canal of *P. scutosa*.

Sensilla basiconica are distributed on both the external galeal surface and the food canal along the whole proboscis. Each sensillum basiconicum consists of a short smooth cone with a sensory pore at the blunt tip, protruding from a round socket (Figure 7).

External sensilla basiconica are irregularly distributed on the galeal surface, and become shorter at the distal region of the proboscis (Figure 7A,B,D,E). *S. trifolii* bears significantly longer and wider external sensilla basiconica on Zone 1 and 2 than *P. scutosa* (Table 2). Males of *S. trifolii* have significantly shorter and wider external sensilla basiconica on Zone 1 than females (Table 3). No significant differences are found in the length or width of external sensilla basiconica between sexes of *P. scutosa* (Table 4).

Internal sensilla basiconica (Figure 7C,F) are regularly spaced in a row in the food canal. The internal sensilla basiconica of *S. trifolii* are noticeably longer and wider than those of *P. scutosa* (Table 2). Males of *S. trifolii* have significantly wider internal sensilla basiconica than females (Table 3). No significant differences are observed in the length and width between sexes of *P. scutosa* (Table 4).

#### 3.2.3. Sensilla Styloconica

**Figure 8:**
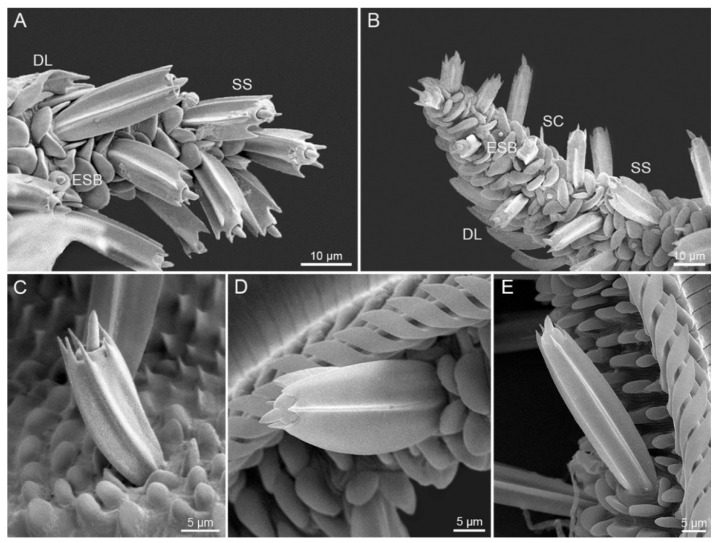
Sensilla styloconica. (**A**) Sensilla styloconica on Zones 2 and 3 of *S. trifolii*; (**B**) sensilla styloconica on Zones 2 and 3 of *P. scutosa*; (**C**,**D**) sensillum styloconicum on Zone 2 of *S. trifolii*; (**E**) sensillum styloconicum on Zone 2 of *P. scutosa.* DL, dorsal legulae; ESB, external sensilla basiconica; SC, sensilla chaetica; SS, sensilla styloconica.

Sensilla styloconica of *S. trifolii* and *P. scutosa* are distributed exclusively on Zone 2 and 3 (Figure 8). Each sensillum styloconicum is composed of a uniporous sensory cone inserted into an elongated stylus, bearing 5–6 longitudinal ribs and protruding from a lotus-shaped base (Figure 8C–F). Sensilla styloconica are progressively shortened toward the tip of the proboscis, generating a brush-shaped appearance on Zone 2 and 3. *S. trifolii* has significantly longer and wider sensilla styloconica than *P. scutosa* (Table 2). Females of *S. trifolii* and *P. scutosa* have markedly wider sensilla styloconica than males (Table 3 and Table 4).

## 4. Discussion

This study made a detailed morphological comparison of proboscis and the associated sensilla between the clover cutworm, *Scotogramma trifolii*, and the spotted clover moth, *Protoschinia scutosa*. The two distant species, *S. trifolii* of Noctuinae and *P. scutosa* of Heliothinae, possess similar proboscis in morphology and structure, both including three sensillum types and three zones (Zone 1–3). The proboscis structure is relatively conservative in Noctuidae, and sensilla morphology may not be useful for comparative studies of nearby species. In Noctuinae, *Mythimna separata* [12] and *Athetis lepigone* [20] were reported to lack Zone 3 at the proboscis tip, while *S. trifolii* possesses Zone 3 (Figure 3D), suggesting a somewhat complex pattern in proboscis morphology. By contrast, both *P. scutosa* and *Helicoverpa armigera* [12] bear Zone 3 at the proboscis tip, which might suggest that Zone 3 is relatively conservative in Heliothinae. Our study indicates that *S. trifolii* of Noctuinae and *P. scutosa* of Heliothinae exhibit significant differences in the dimension of each zone, as well as in the sensilla size. These morphological results probably reinforce the previous conclusion that the proboscis and associated sensilla could provide potential values in the systematic and phylogenetic analyses at the subfamily level within Noctuidae.

The proboscises of *S. trifolii* and *P. scutosa* are tightly coiled in the resting position, and the cuticular processes might help maintain the resting position [36]. The proboscis extends to a relatively straight shape when feeding, producing converse effects on the antero-dorsal and postero-ventral surfaces [37]. Two groups of maxillary muscles, galeal and stipital muscles, are used to control the proboscis movement, as reported in other lepidopterans [38]. The proboscis uncoiling is caused by the increase of hemolymph pressure of stipites forcing hemolymph into galeae [39]. By contrast, proboscis recoiling is supported by the elasticity of the galeal cuticle [1,40] and the contraction of the oblique longitudinal intragaleal muscles [41]. The proboscis coiling starts at the tip and progresses toward the base. The diameter of the spiral widens due to its elastic properties until the proboscis props itself against the ventral side of the head [36]. This elastic effect combined with the tightly linking cuticular processes may hold the resting position of the proboscis. Coiling and bending not only help package and protect the proboscis, but also provide additional means to optimize fluid intake [42].

The lepidopteran proboscis is functionally divided into a hydrophobic and a hydrophilic region [35]. Zone 1 with a closed interlocking structure is considered to be a hydrophobic non-drinking region, whereas Zones 2 and 3 are regarded as hydrophilic drinking regions [22]. The hydrophobic region bears overlapping dorsal legulae and small interlegular spaces with a large proportion of the circumference covered with hydrophobic microbumps [43]. The hydrophilic regions of *S. trifolii* and *P. scutosa* account for 5–20% of the total length of the proboscis [44,45], and bear slits at the tip which actively lead fluid into the food canal [46,47]. Liquid absorption was originally explained by the drinking-straw model of the proboscis [48]. However, recent studies have revealed that Lepidoptera pull fluid from the porous surface into the food canal by capillary action via the interlegular spaces and the spaces between the hydrophilic sensilla styloconica and the pressure gradient created by the sucking sump [45,49]. Fluid uptake with the proboscis is mainly comprised of four steps: wetting, dewetting, absorbing, and pumping [50,51]. Many physical determinants represent the fundamental architecture of the proboscis affecting fluid uptake [52]. For example, the absorption efficiency is affected by increased resistance from tapering of the food canal in the drinking region and the viscous resistance of the membranes spreading along the food canal [46,49]. *S. trifolii* possess significantly longer Zones 2 and 3 than *P. scutosa*, probably suggesting a better ability of fluid absorption of *S. trifolii*.

Three sensillum types on the proboscis of *S. trifolii* and *P. scutosa* play different roles in feeding behavior. The aporous sensilla chaetica are considered to be mechanosensitive [16,53,54], probably involved in detecting the depth of proboscis insertion during flower probing and monitoring the coiling status in the resting position [36]. The external sensilla basiconica are uniporous and act as chemoreceptors (taste and gustatory receptors) [16,53,55]. The internal sensilla basiconica on the food canal could provide information on the passage from nectar to the pharynx, as reported in butterfly species [16,56]. The sensilla styloconica of both species are located exclusively at the proboscis tip in a dense brush-like configuration, similar to those of *Hypsoropha hormos* [57], *Choristoneura fumifernana* [55], and *Laspeyresia pomonella* [58]. In most lepidopterans, each sensillum styloconicum bears a single pore at the apex of the cone, and is inferred to be derived from sensillum basiconicum [16]. The uniporous sensilla styloconica function as contact chemo-mechanoreceptors to explore the corolla entrance of the flower and taste nectar [59,60]. Each sensillum styloconicum is reported to be innervated by dendrites from receptor cells [61], and sensitive to sugars, nicotine, and other substances [62,63].

The noctuid moths are mostly flower-visiting pollinators, and use the proboscis tip to pollinate [64,65,66]. These two species, *S. trifolii* and *P. scutosa*, are most likely to be flower-visiting noctuids, and characterized by elongated sensilla styloconica and modified dorsal legulae on the proboscis, as reported in previous studies [15,19,20,67,68]. The conclusion is also reinforced by the presence of floral pollen at the proboscis tip of *P. scutosa* (Figure 2). The morphological adaptability of the proboscis is mainly manifested in its length and the tip region with specially shaped sensilla and cuticular structures [14,23,69,70]. The proboscis morphology of flower visitors generally varies according to the diverse floral structures [22]. The longer and more finely pointed the proboscis, the more readily accessible is the corolla for feeding and nutrient acquisition [71]. Compared with *S. trifolii*, *P. scutosa* possess a long proboscis with a narrow tip, which might suggest that *P. scutosa* could better stretch into a long and slender flower tube than *S. trifolii*. In the potential flower visiting noctuids, Zone 3 is present on the proboscis tip of *S. trifolii* and *P. scutosa*, and absent on the proboscis tip of *M. separata* and *A. lepigone* [12,20,72]. The presence of Zone 3 might have adaptive value in facilitating the proboscis to enter narrow floral corollas, as described in Lehnert et al. [22]. Further studies may be needed to explore the function of Zone 3 in the evolution of adult feeding habits of noctuid species.

## 5. Conclusions

This study shows that the proboscises of *S. trifolii* and *P. scutosa* are structurally similar, both composed of two tightly linked maxillary galeae that enclose the central food canal by dorsal and ventral legulae. The proboscis was structurally delineated into three zones (Zone 1–3), and possesses three types of sensilla, sensilla chaetica, basiconica, and styloconica. However, *S. trifolii* and *P. scutosa* exhibit significant differences in the length of the proboscis and each zone, as well as in the dimension of each sensillum type. The presence of floral pollen, in combination with the characteristics of the distal proboscis indicates that *S. trifolii* and *P. scutosa* are very likely flower visitors. These findings provide a morphological basis to better understand the feeding mechanisms of both species.

## Figures and Tables

**Table 1:** Length of proboscis and width of food canal of *Scotogramma trifolii* and *Protoschinia scutosa*.

Type	Length/Width (μm)		*t*-Test
	*S. trifolii*	*P. scutosa*	
Proboscis	7478.07 ± 159.76 (14)	7858.15 ± 157.38 (14)	NS
Food canal	61.58 ± 0.92 (28)	59.42 ± 0.66 (28)	*
Zone 1	6426.14 ± 165.60 (14)	7157.51 ± 156.61 (14)	*
Zone 2	1049.68 ± 36.54 (14)	816.44 ± 25.68 (14)	*
Zone 3	35.03 ± 1.49 (8)	39.03 ± 0.65 (14)	*

Note: Data are presented as Mean ± SE (*n*); *n*, sample size; NS, nonsignificant differences; * indicates *p* < 0.05 in the independent samples *t*-test.

**Table 2:** Length and basal width of each sensillum type of *Scotogramma trifolii and Protoschinia scutosa*.

Type	Length (μm)		*t*-Test	Basal Width (μm)		*t*-Test
	*S. trifolii*	*P. scutosa*		*S. trifolii*	*P. scutosa*	
SC	46.41 ± 1.93 (140)	60.20 ± 2.13 (140)	*	3.23 ± 0.06 (140)	3.11 ± 0.06 (140)	NS
SST	37.28 ± 0.52 (280)	30.90 ± 0.48 (280)	*	8.26 ± 0.09 (280)	7.50 ± 0.09 (280)	*
ESB (Zone 1)	9.67 ± 0.28 (140)	6.81 ± 0.21 (140)	*	2.37 ± 0.04 (140)	2.15 ± 0.05 (140)	*
ESB (Zone 2)	6.67 ± 0.43 (28)	4.41 ± 0.30 (28)	*	2.14 ± 0.07 (28)	1.87 ± 0.08 (28)	*
ISB	8.01 ± 0.30 (50)	6.82 ± 0.29 (50)	*	2.46 ± 0.05 (50)	2.49 ± 0.05 (50)	NS

Note: Data are presented as Mean ± SE (*n*); *n*, sample size; NS, nonsignificant differences; * indicates *p* < 0.05 in the independent samples *t*-test. Abbreviations: SC, sensilla chaetica; SST, sensilla styloconica; ESB, external sensilla basiconica; ISB, internal sensilla basiconica.

**Table 3:** Length/Width of proboscis and and each sensillum type of *Scotogramma trifolii* males and females.

Types	Length (μm)		*t*-Test	Basal Width (μm)		*t*-Test
	Male	Female		Male	Female	
Proboscis	7583.81 ± 640.84 (7)	7372.33 ± 580.87 (7)	NS	–	–	–
Zone 1	6570.53 ± 253.31 (7)	6281.75 ± 218.44 (7)	NS	–	–	–
Zone 2	1008.78 ± 46.84 (7)	1090.58 ± 55.07 (7)	NS	–	–	–
Zone 3	33.67 ± 2.01 (4)	36.39 ± 2.24 (4)	NS	–	–	–
SC	44.24 ± 3.33 (70)	48.58 ± 2.82 (70)	NS	3.44 ± 0.09 (70)	3.03 ± 0.06 (70)	*
SST	36.43 ± 0.63 (140)	38.13 ± 0.82 (140)	NS	9.10 ± 0.13 (140)	7.43 ± 0.08 (140)	*
ESB (Zone 1)	8.99 ± 0.24 (70)	10.36 ± 0.49 (70)	*	2.50 ± 0.06 (70)	2.24 ± 0.05 (70)	*
ESB (Zone 2)	6.39 ± 0.39 (14)	6.94 ± 0.78 (14)	NS	2.27 ± 0.08 (14)	2.01 ± 0.10 (14)	NS
ISB	7.54 ± 0.46 (25)	8.49 ± 0.36 (25)	NS	2.64 ± 0.07 (25)	2.28 ± 0.06 (25)	*

Note: Data of proboscis sensilla are presented as Mean ± SE (*n*); *n*, sample size; NS, nonsignificant differences; * indicates *p* < 0.05 in the independent samples *t*-test; –, present but not counted. Abbreviations: SC, sensilla chaetica; SST, sensilla styloconica; ESB, external sensilla basiconica; ISB, internal sensilla basiconica.

**Table 4:** Length/Width of proboscis and each sensillum type of *Protoschinia scutosa* males and females.

Types	Length (μm)		*t*-Test	Basal Width (μm)		*t*-Test
	Male	Female		Male	Female	
Proboscis	8065.79 ± 195.46 (7)	7650.50 ± 234.00 (7)	NS	–	–	–
Zone 1	7362.79 ± 186.98 (7)	6952.23 ± 239.31 (7)	NS	–	–	–
Zone 2	865.20 ± 39.11 (7)	767.67 ± 23.16 (7)	NS	–	–	–
Zone 3	38.83 ± 0.64 (7)	39.24 ± 1.18 (7)	NS	–	–	–
SC	59.02 ± 3.19 (70)	61.38 ± 2.83 (70)	NS	3.27 ± 0.09 (70)	2.94 ± 0.09 (70)	*
SST	31.16 ± 0.65 (140)	30.64 ± 0.72 (140)	NS	7.82 ± 0.13 (140)	7.18 ± 0.13 (140)	*
ESB (Zone 1)	6.54 ± 0.27 (70)	7.16 ± 0.31 (70)	NS	2.22 ± 0.07 (70)	2.10 ± 0.07 (70)	NS
ESB (Zone 2)	4.28 ± 0.40 (14)	4.54 ± 0.46 (14)	NS	1.89 ± 0.1 (14)	1.86 ± 0.09 (14)	NS
ISB	6.80 ± 0.45 (25)	6.85 ± 0.39 (25)	NS	2.48 ± 0.09 (25)	2.49 ± 0.07 (25)	NS

Note: Data of proboscis sensilla are presented as Mean ± SE (*n*); *n*, sample size; NS, nonsignificant differences; * indicates *p* < 0.05 in the independent samples *t*-test; –, present but not counted. Abbreviations: SC, sensilla chaetica; SST, sensilla styloconica; ESB, external sensilla basiconica; ISB, internal sensilla basiconica.

## Data Availability

The data presented in this study are available on request from the corresponding author.
